# Altered resting state EEG microstate dynamics in acute concussion in adolescents

**DOI:** 10.1038/s41598-026-37259-7

**Published:** 2026-02-02

**Authors:** Sahar Sattari, Samir Damji, Julianne McLeod, Maryam S. Mirian, Lyndia C. Wu, Naznin Virji-Babul

**Affiliations:** 1 School of Biomedical Engineering, University of British Columbia, Vancouver, British Columbia Canada; 2 Department of Neuroscience, University of British Columbia, Vancouver, British Columbia Canada; 3 Department of Rehabilitation Sciences, University of British Columbia, Vancouver, British Columbia Canada; 4 Pacific Parkinson’s Research Centre, University of British Columbia, Vancouver, British Columbia Canada; 5 Djavad Mowafaghian Centre for Brain Health, University of British Columbia, Vancouver, British Columbia Canada; 6 Department of Mechanical Engineering, Faculty of Medicine, University of British Columbia, Vancouver, British Columbia Canada; 7 Department of Physical Therapy, Faculty of Medicine, University of British Columbia, Vancouver, British Columbia Canada

**Keywords:** Biomarkers, Neurology, Neuroscience

## Abstract

Concussion is a global health concern; however, the neurophysiological underpinnings remain poorly understood, with a lack of clinically relevant, objective brain-based approaches. We investigated the potential of EEG microstate analysis to characterize alterations in brain activity post-concussion. We applied a modified k-means clustering algorithm to multi-channel resting-state EEG data, comparing participants within 2 weeks post-concussion to age- and sex-matched healthy controls. EEG topographical maps were classified into seven clusters, with each cluster represented by canonical microstates (A–G). Average duration, occurrence rate, and time coverage for each microstate were extracted and compared between groups. Multiple correlation analyses were performed between microstate measures and symptom severity within the concussed group. The concussed group showed a statistically significant reduction in the duration of microstate E and the duration, occurrence, and time coverage of microstate G compared to controls. The occurrence rate and time coverage of microstate C were significantly higher in the concussed group. There was a positive correlation between symptom severity and the duration and occurrence of microstate E; however, this did not reach statistical significance. Together, these findings indicate that acute concussion disrupts the dynamic interactions of large-scale brain networks and suggest that EEG microstate analysis applied to resting-state data may provide a potential biomarker of injury.

**Supplementary Information:**

The online version contains supplementary material available at 10.1038/s41598-026-37259-7.

## Introduction

The neurophysiological and neurobiological changes due to concussion or mild traumatic brain injury (mTBI; hereafter used synonymously with concussion) are not well understood. Concussion results from biomechanical forces exerted on the skull and the subsequent shearing and stretching of brain tissue; although this injury is particularly prevalent, concussion diagnosis remains a challenge due to its largely subjective nature and the absence of definitive clinical tests^1^. There is a need for objective, brain-based diagnostic methods that can reliably indicate the occurrence of a concussion^2,3^. The dynamic and complex nature of concussion injury is of particular concern in adolescents due to the complexity and heterogeneity of neurodevelopment.

In Canada, concussion incidence is highest among adolescents aged 12 to 19 compared to other age brackets across the lifespan; this result is corroborated by a similar epidemiological investigation of the U.S. population^4,5^. It should be noted that most concussion injuries in this age group are sustained during sport (hockey, rugby, and football in particular) and other physical activities^4^.

Adolescents are particularly vulnerable to the neuropsychological consequences that stem from sustaining a concussion. A growing number of structural and functional brain imaging studies indicate that adolescence is an extremely dynamic period of brain development^6,7^, and an mTBI superimposed on a rapidly developing brain and body undergoing puberty reportedly leads to more severe and persistent symptoms in comparison to younger children and adults^8,9^.

The current methods used in concussion detection, monitoring, and return-to-play clearance for adolescent athletes are suboptimal; these tools tend to lack either objectivity, utility, or feasibility for this population^10,11^. Functional brain imaging studies on concussion to date have begun to provide some clues regarding potential biomarkers of concussion. Resting-state fMRI studies have examined the disruptions to brain functional connectivity caused by concussion. This research has shown alterations in both dynamic (i.e., spending less time in a frontotemporal default mode/limbic brain state) and static measures of functional connectivity (i.e., altered interhemispheric connectivity, as well as hyperconnected frontal nodes and hypoconnected posterior nodes in the salience and fronto‑parietal networks) relative to healthy control subjects^12,13^.

While these findings are informative, the feasibility of performing multiple MRI tests on young athletes as an objective diagnosis/recovery assessment is not ideal and often not available in remote regions. Portable and feasible neuroimaging techniques are required that are sensitive to changes in the brain post-impact. Electroencephalography (EEG) has the potential to serve as an objective diagnostic tool and has been extensively studied in recent years for this purpose.

EEG microstate analysis describes the discrete functional cortex-wide states that occur in the resting-state brain. During the resting state, the pattern of scalp potentials remains quasi-stable for approximately 30–120 milliseconds before transitioning to another topographical pattern and is thought to reflect the rapid reconfiguration of large-scale neural networks^14–16^. These microstates show high reliability^17^. This resting-state analysis method provides a lens for understanding altered dynamics of large-scale brain networks, rather than isolated features of the EEG signal^18^. EEG microstates have been shown to have strong associations with large-scale fMRI resting state brain networks (RSNs)^19,20^. Additionally, EEG microstates capture subtle temporal dynamics about these functional brain areas and networks that cannot be captured with resting-state fMRI alone, given the limited temporal resolution of the BOLD signal. To our knowledge, EEG microstate analysis has been applied by only one group to study concussion^21^. However, this group investigated adults (mean age of 40 yrs) who had sustained a concussion several years prior (0.3–16 yrs), and there was no healthy control comparison group. In this chronic adult group with chronic neurophysiological impairment the duration of the four canonical microstates (A–D) was negatively correlated with a neuropsychological impairment index. Only four microstate classes were investigated, and only the average duration of each microstate was investigated. To our knowledge, this is the first study to examine whether EEG microstate dynamics are altered in adolescents during the acute phase of mTBI injury.

In the present study, we investigate acute-phase changes in EEG microstate features in a cohort of adolescent males, with the primary aim of enhancing our current understanding of neural dynamic changes associated with concussion. We hypothesized that mTBI will be associated with differences in microstate sequences as an indicator of acute changes in temporal dynamic interplay of large-scale brain networks in comparison to healthy controls, and that these changes will be correlated with concussion symptoms. This pilot study is part of a larger project that aims to identify potential microstate markers of concussion to enhance the objectivity and reliability of concussion diagnoses and recovery assessments.

A preliminary version of this work was previously made available as a preprint^22^. The current manuscript includes updated analyses and results.

## Materials and methods

### Participants

Thirty-four right-handed male athletes aged 10–18 years were recruited for this study. Twenty participants had no history of concussion, and 14 participants received a physician-confirmed diagnosis of concussion. Concussed participants were within two weeks of injury and met the consensus diagnostic criteria for concussion in sport^23^. Exclusion criteria for all participants included the presence of focal neurological deficits, underlying neurological pathology or the use of prescription medications for neurological or psychiatric conditions.

Symptom number and severity in concussed participants were evaluated using the Sports Concussion Assessment Tool 3 (SCAT3). The SCAT3 assesses 22 symptoms rated on a scale from 0 (none) to 6 (severe). A total symptom severity score was calculated by adding all the ratings for a maximum score of 132. Details are presented in Table 1.

The study was approved by the University of British Columbia Clinical Research Ethics Board (Approval number: H17-02973) in accordance with the Helsinki Declaration. All research was performed in accordance with relevant guidelines/regulations. All participants provided written assent, and their parents/guardians gave written informed consent as per the guidelines of the Human Ethics Review Board of the University of British Columbia. Participant recruitment began on March 15, 2019, and is ongoing. The current ethics protocol was renewed and is valid until October 24, 2025.

### EEG data collection and preprocessing

Five minutes of eyes-closed, resting-state EEG data were collected from all individuals using a 64-channel HydroCel Geodesic Sensor Net (EGI, Eugene, OR). After obtaining assent and informed consent, participants were seated in an experimental room with controlled lighting levels and fitted with the EEG cap. They were instructed to minimize movement and remain seated with their eyes-closed. Before initiating the data collection, the electrode-scalp resistance was checked to be below 50 k$\:{\Omega\:}.\:\:$ This is the standard and recommended practice for electrode systems such as EGI, which consist of active electrodes and a high input impedance amplifier to achieve a high-quality signal.

The signals were referenced to the vertex (Cz) and recorded at a sampling rate of 250 Hz. The collected EEG data were imported into Python for preprocessing. The initial step involved re-referencing the data from the current reference (Cz) to the average of all channels. A zero-phase 4th -order Butterworth high-pass filter at 1 Hz was then applied, followed by a 4th -order low-pass Butterworth filter at 40 Hz and a 60 Hz notch filter. This frequency range was chosen due to excessive noise at frequencies above 40 Hz, which interfered with subsequent preprocessing steps. We then used *pyprep* to label bad channels using the correlation between neighboring channels with default threshold of 0.4^24^. On average $\:3.1$ channels were identified as bad channels and were interpolated using spherical spline interpolation^25^ integrated in MNE preprocessing package in Python^26^. No data segments were rejected during preprocessing.

Independent component analysis (ICA) was performed using the *infomax* algorithm and the MNE preprocessing package in Python^27^. To identify and remove noise-contaminated components, we employed the *ICLabel* algorithm, an automated labeling tool for independent components^27^. The method classifies the components using scalp topography, power spectral density and time domain features of ICs^28^. We retained only those components that the algorithm labeled as *“Brain”* (i.e. probability of the component presenting brain source was higher than artifactual source). These retained components were then confirmed by manual inspection. Between 2 and 9 components were removed per participant (mean: controls = 5.9; mTBI = 4.0). The number of components removed did not differ significantly between groups (p > 0.1).

### Microstate analysis

For the microstate analysis, we utilized the *Pycrostates* package in Python^29^. Global Field Potential (GFP) peaks were extracted from individual data sets using the functions available in the *Pycrostates* package. This process includes calculating the standard deviation of all channel values at each time point, generating a time series of GFP values. Subsequently, peaks in this time series were identified using the *Scipy* integrated function, with the minimum distance between peaks set to the default value of 2 samples. We identified the minimum number of GFP peaks across all subjects. To ensure equal contribution from each participant to the global clustering process, the number of GFP peaks was standardized across the cohort. Specifically, the minimum number of GFP peaks observed in any individual dataset was identified. For all remaining participants, GFP peaks were randomly subsampled to match this minimum count. This procedure minimized clustering bias arising from inter-individual differences in peak density. The resulting GFP peaks were then clustered using a modified k-means algorithm, consistent with established EEG microstate methodologies.

Previous studies have typically used between 4 and 7 microstates for EEG microstate analysis, but there is currently no consensus on the optimal number of classes to use^30,31^. While early research focused on 4 canonical microstate templates (A to D), more recent evidence suggests that 7 distinct templates (A to G) better capture the diversity of spontaneous electrophysiological topographies observed in resting-state EEG^31^. Our labeling system is based on the recent work by Tarailis et al.^30^, who proposed a new microstate labeling system to facilitate cross-study comparisons. Within this system, four classical microstates are labelled as A to D and an additional three (E to G) are labelled based on the frequency of these states from a comprehensive review of studies that have reported the additional microstates.

We selected a seven-class solution as it provided the best fit to the cohort’s data by maximizing Global Explained Variance (GEV) and closely aligning with topographical patterns reported in the literature^31^. Further details are provided in the Supplementary materials.

We aligned the 7 microstates with the established microstate labels (A to G) based on the topographical shapes of the states. We then assigned each time point of the original dataset to one of the 7 microstates, using a “winner takes all” strategy, a smoothing parameter of 6 samples (24 ms), and correlation between topographical maps as the similarity measure. This involved correlating each time point with each microstate and assigning the time point to the state with the highest correlation, taking the smoothing parameter into account but not polarity. The smoothing was applied to reduce the influence of rapid fluctuations, potentially caused by artifacts, and to prevent false identification of microstate changes.

**Fig. 1:**
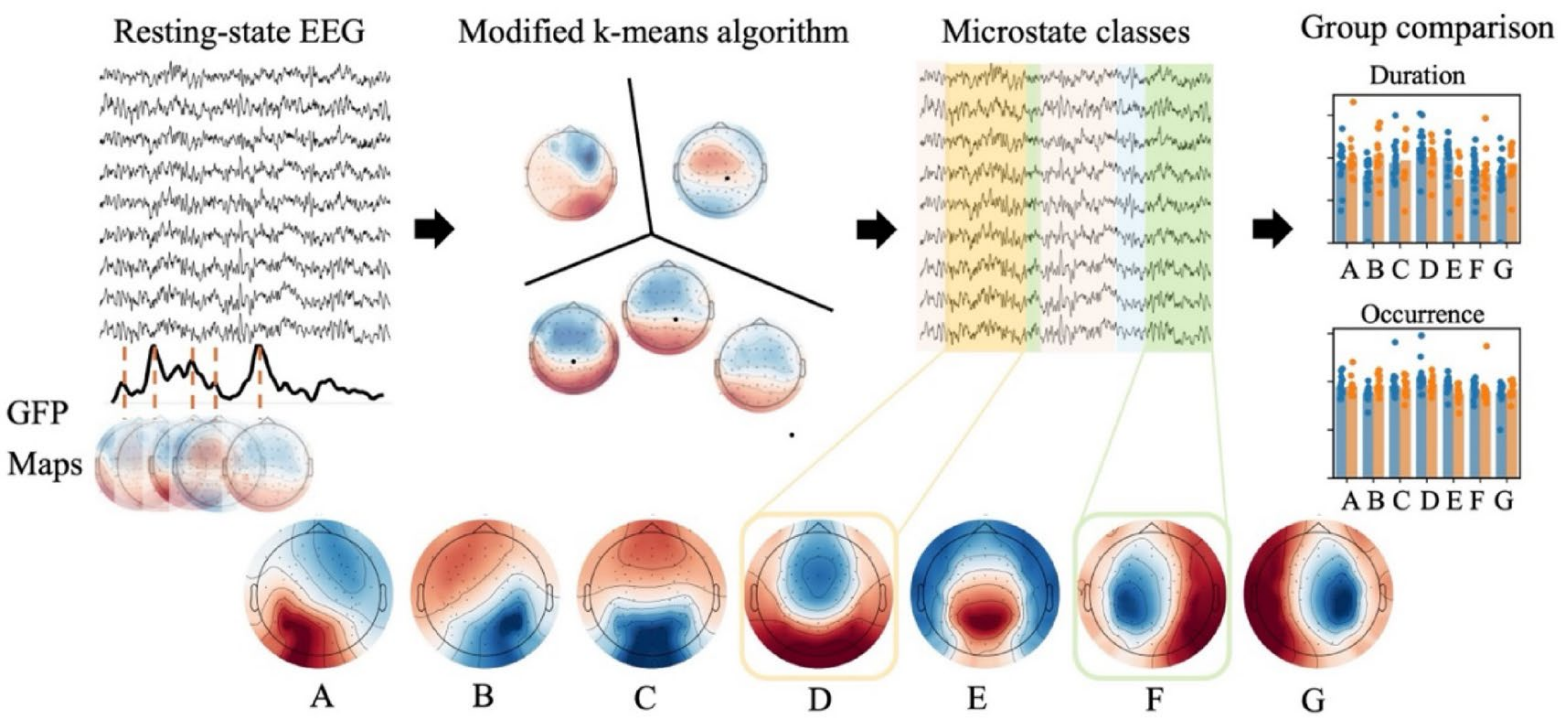
Study pipeline for microstate extraction from resting-state EEG to differentiate between groups. The Global Field Power (GFP) time series is computed, and microstate maps are identified by clustering maps from the local peaks of the GFP. Seven distinct microstates are identified. The maps are labeled based on their shape and the labeling method introduced in^31^. The original EEG time series is backfitted to these microstates using a “winner-takes-all” algorithm. Key metrics such as microstate duration, occurrence rate, and time coverage are then extracted for group comparison.

Three measures were extracted from each individual’s microstate sequence: average duration, occurrence rate, and time coverage. First, the average duration of each microstate was calculated by determining how long the microstate remained unchanged in each sequence. These individual durations were averaged for each subject, and group-level means and standard deviations were extracted. The same method was applied to calculate the occurrence rate of each microstate (i.e., the number of occurrences per second) and the time coverage (i.e., the proportion of time each microstate occupied relative to the total recording time). Lastly, we also extracted the average transition rate per second between microstates for each individual, to evaluate whether the stability of staying in one state or frequent transitions between states may be a feature of mTBI. Figure 1 presents the pipeline.

### Statistical analysis of microstate features

We used permutation statistics for all comparisons. For each feature, we conducted seven tests (one corresponding to each microstate). We applied a significance level of $\:\frac{0.05}{7}\:$according to the Bonferroni method to adjust for multiple comparisons. During each test, we randomized the subjects from both groups and generated new groups of the same size as the original ones. We then conducted a t-test on these newly formed random groups. If the t-value from the random groups exceeded that of the original groups, we added the error rate by one. This procedure was repeated 10,000 times. The total error rate was then calculated and divided by the number of permutations. Features and microstates falling below the adjusted significance threshold are reported.

We performed multiple regression analyses to examine the relationships between microstate features and clinical symptom measures derived from the SCAT3. Mean microstate duration and occurrence rate for each microstate were entered as independent variables, while symptom number and overall symptom severity served as dependent variables. To control for multiple comparisons, the significance threshold was adjusted to *p* = 0.007. Time coverage was not included in the regression models, as it can be derived from the average duration and occurrence rate; therefore, inclusion of time coverage would be redundant if significant associations were observed for these primary features.

## Results

### Demographics

All concussed participants were tested between 1 and 2 weeks of injury. Data from two acutely concussed males were removed due to excessive noise remaining on EEG after preprocessing, leaving a final sample of 32 male adolescent athletes between the ages of 13 and 18 (Table 1). No significant age difference was observed between groups (*p* = 0.1). The total number of symptoms and symptom severity from the SCAT3 are reported for 8 participants. All the concussed participants met the diagnostic criteria for concussion, were symptomatic at the time of testing and exhibited a minimum of 4 and as many 18 symptoms (out of maximum of 22). The most common symptoms were difficulty concentrating/remembering, dizziness, feeling like in a “fog”, sensitivity to light, “don’t feel right,” fatigue, and irritability. The severity of symptoms ranged between 2 and 63 (out of a maximum of 132).

**Table 1:** Demographic characteristics of study Participants.

Demographic Information	Controls (*N* = 20)	Concussed (*N* = 12)
Age (Years, min: max)	16 (14:18)	15.1(13:18)
Time since concussion		Within 2-Weeks
SCAT (# of symptoms, SD)		9.1 (6.8) (*N* = 8)
SCAT (Symptom severity, SD)		21 (20.4) (*N* = 8)

### Microstate duration, occurrence rate and time coverage

**Fig. 2:**
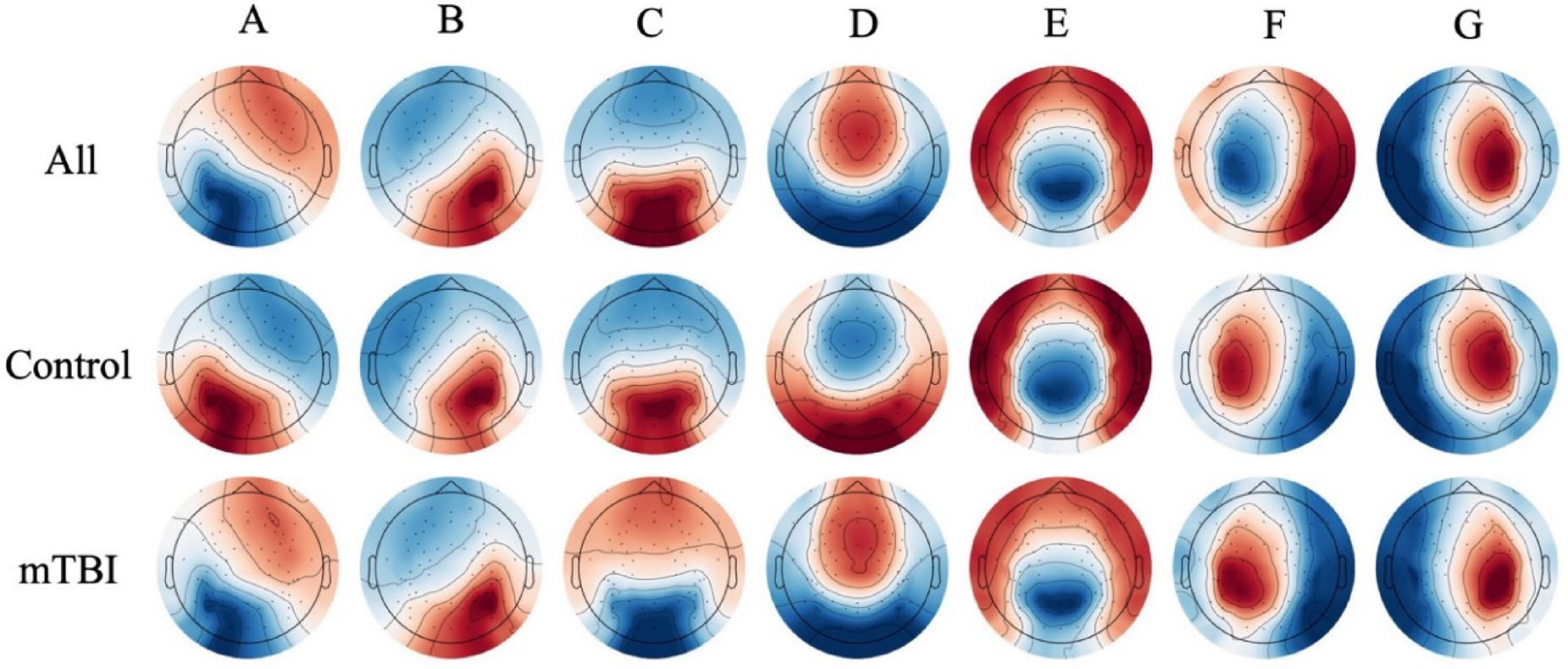
Topographies of the seven extracted microstates. The first row shows the maps generated using data from both groups combined. The second and third rows show the maps generated separately for the control and concussed groups, respectively. The high similarity across the three sets (as shown in the Supplementary materials) supports the use of the combined-group maps for all subsequent analyses. As is standard in microstate analysis, topographic polarity is not considered.

Seven microstates with topographies similar to those identified in the meta-analysis by Koenig et al. (2024) were extracted (Fig. 2). Three sets of maps were generated: one using data from both groups combined, and one for each group separately. Due to the high similarity between the group-specific maps and to reduce the risk of Type I error^32^, the rest of the analyses were performed using the maps generated from the combined group data.

**Fig. 3:**
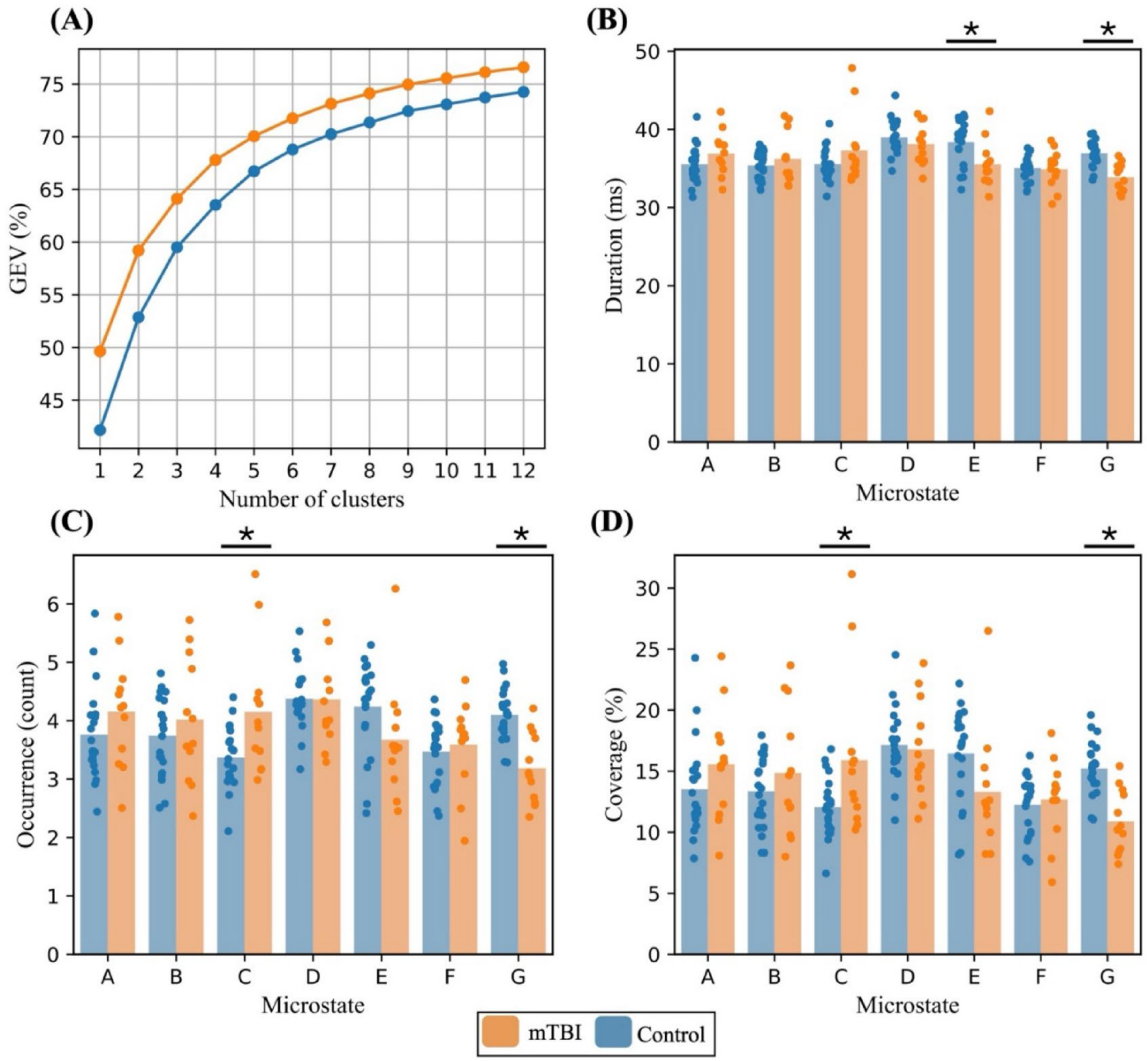
Microstate global variance, and comparative metrics in healthy vs. concussed participants. (**A**) Global explained variance (GEV) as a function of the number of clusters, shown separately for mTBI (orange) and control (blue) groups. (**B**), (**C**) and (**D**) Bar plots representing the three key microstate measures: average duration, average occurrence rate, and time coverage, respectively. Healthy (blue, *n* = 20) and concussed (orange, *n* = 12) groups are compared, with dots representing individual data points. Significant differences in mean duration, occurrence rate, and time coverage of microstates C, G and E were observed using adjusted significance threshold of $\:0.007$. Overall, microstate E showed a significantly shorter duration in the concussed group compared to controls. Microstate C showed significantly higher occurrence and time coverage in the concussed group, and microstate G showed significantly lower duration, occurrence, and time coverage $\:(p\:<\:0.001).$.

The variance explained by the set of seven microstate series was calculated for individual datasets following the backfitting process (Fig. 3A). The total global explained variance (GEV) was $\:71.61\%$. The analysis revealed several significant differences. First, the duration of microstate E was statistically significantly shorter in the concussed group compared to controls ($\:p < 0.001;\:Cohen^{\prime}sd = 1.02)$. In addition, the duration, occurrence and time coverage of microstate G was significantly reduced ($\:p < 0.001;\:Cohen^{\prime}s\:d = 1.88,\:1.80,\:1.85\:$respectively) compared to the control group. Lastly, there was a significant increase in occurrence and time coverage of microstate C in concussed group compared to controls ($\:p < 0.001;\:Cohen^{\prime}s\:d = 0.94,\:0.81$ respectively) (Fig. 3B, C and D).

### Microstate transition rate

**Fig. 4:**
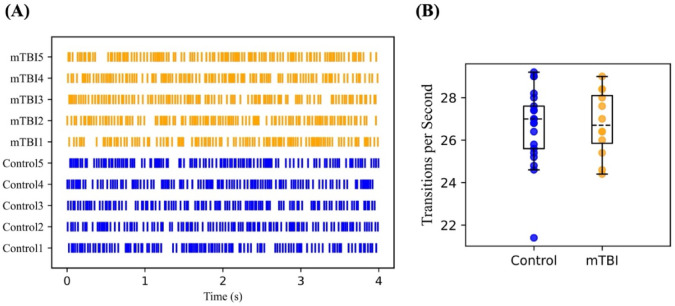
Rate of transitions in microstate sequences. (**A**) Shows a spike for each transition from one microstate to another within a 4-second time window for example subgroups. (**B**) Shows a box plot comparing the transition rates per second between the control and mTBI groups, with individual data points representing the average number of transitions per second for each participant in the cohort. The difference between the two groups was not statistically significant.

Transitions were recorded for each second of the data. The total number of transitions per second was averaged over the entire data length for each individual. We observed lower average in transition rate in concussed group. However, this difference was not statistically significant (Fig. 4).

### Concussion symptoms related to microstate features

**Fig. 5:**
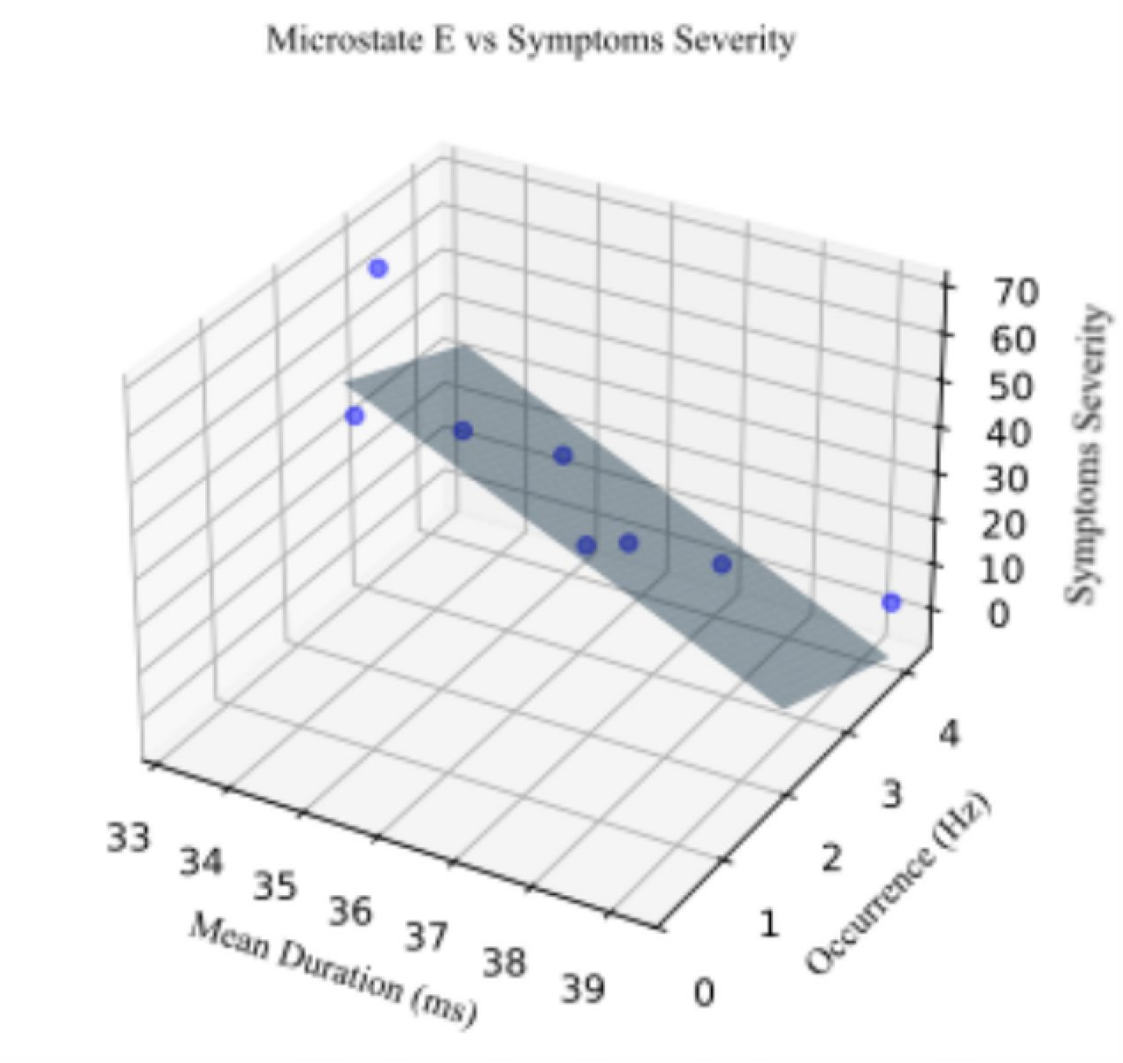
Multiple regression model. The average duration and occurrence rate of microstate E are used as independent variables, and symptom severity is used as the dependent variable. Data from eight concussed participants were included in this model due to the absence of SCAT information from other concussed individuals. The model demonstrates a strong linear relationship between microstate E features and symptom severity $\:({r}^{2}\:=\:0.71);$ however, this association does not reach significance under the adjusted threshold $\:(p\:=\:0.04$).

Among the eight concussed athletes who completed the SCAT, microstate E had a negative linear relationship with the SCAT symptom severity measure $\:(p\:=\:0.04,\:F\:=\:5.98,\:\:{r}^{2}=0.71).$ This model indicates that increased symptom severity is associated with shorter duration and lower occurrence rates of microstate E. However, when applying a Bonferroni-adjusted significance threshold of $\:p<0.007$ to account for multiple comparison, this result does not reach statistical significance (Fig. 5).

## Discussion

In the present study, we applied EEG microstate analysis to explore changes associated with acute sport-related concussion in a small cohort of adolescent male athletes. Despite the small sample size, these preliminary findings reveal distinct alterations in resting-state EEG microstate dynamics in adolescent male athletes during the acute phase of sport-related concussion. Our main finding was a significant reduction in the duration of microstate E. Microstate E is characterized by a topography displaying a centro-parietal maximum^30^ and has been linked to activity in the medial prefrontal cortex, dorsal anterior cingulate cortex, insula and superior frontal gyrus^33,34^. It is associated with the salience network (SN), suggesting impaired attentional engagement and network switching, functions that are crucial for environmental awareness, detecting and filtering salient stimuli and coordinating other brain networks to guide behavior. While exploratory, this reduction correlated with symptom severity, highlighting the potential of microstate E as a biomarker for post-concussive cognitive dysfunction, possibly reflecting real-time symptom burden^35–37^.

In parallel, the decreased occurrence and time coverage of microstate G implicate the sensorimotor network, aligning with subtle motor processing changes often reported after concussion. Microstate G with a topography with right-lateralized activity has been linked to activity in the right inferior parietal lobe, superior temporal gyrus and the cerebellum^33^. The link between this microstate and the activity of somatosensory network and cerebellum can also indicate a relationship between concussion and cerebellar activity, which has been reported multiple times in the literature (e.g^38,39^).

Conversely, microstate C showed an increase in occurrence and time coverage in the concussed group with a topography displaying symmetric anterior-to-posterior configuration. This microstate is associated with activity in the precuneus, posterior cingulate cortex and angular gyrus^33^ and has been linked to self-experience, spontaneous cognition and mind wandering^40^ all functions associated in part with the default mode network (DMN). The observed increase may reflect a compensatory recruitment or hyperconnectivity in response to disruption in higher order networks related to the function of the DMN, often reported in the literature. In fact, Liu et al.^41^ have shown that the impaired functional coupling between the DMN and salience networks appears to underlie many of the cognitive impairments in acute mTBI. These changes may reflect a more distracted or unfocused cognitive state, which is often reported in individuals with concussion.

We also analyzed the number of transitions between microstates for each participant. This measure reflects the brain’s ability to switch between different functional states, which is crucial for maintaining efficient cognitive functioning. In a previous fMRI study, we observed reduced transitions between functional states in individuals with a concussion, suggesting cognitive rigidity^42^. However, this observation did not reach statistical significance in the current study. To draw more definitive conclusions, future studies should explore this further with larger sample sizes and greater statistical power.

Taken together, our exploratory results highlight the potential utility of EEG microstate analysis as a sensitive approach for detecting subtle yet meaningful alterations in brain function. The portability of EEG combined with microstate analysis suggests that it could be used as a practical tool for rapid concussion screening in sports or remote settings, warranting further validation in larger cohorts.

### Limitations and future perspectives

Our study results have limited generalizability as we were only able to include a relatively small and homogeneous cohort of male adolescent participants. Future studies should build on these preliminary findings by performing microstate analysis in a larger and more diverse sample and using a longitudinal design to account for within-subject variabilities. An additional limitation is that only 8 of the 12 concussed participants completed the SCAT3, reducing the statistical power of the correlation analysis between symptom quantity/severity scores and microstate features.

Despite these limitations, this study provides a foundation for future research. Longitudinal tracking of athletes from the acute phase through full recovery would enable assessment of the predictive and prognostic utility of EEG microstates. Moreover, microstate analysis holds potential to enhance clinical management: currently, return-to-play decisions rely heavily on subjective symptom reporting, which may be underreported. Objective brain-based measures could therefore offer a more reliable indicator to support clinical decision-making.

## Conclusion

Our results, though exploratory, suggest that resting-state EEG combined with microstate analysis could offer a non-invasive, real-time neural marker of concussion severity. By examining the temporal dynamics of EEG microstates, we identified significant and notable disruptions in the interactions of large-scale brain networks in acutely concussed adolescent male athletes compared to matched controls. Microstate analysis offers a readily accessible and reproducible method for capturing whole-brain activity patterns with high temporal precision which are critical for detecting subtle functional alterations following concussion.

## Supplementary Information

Below is the link to the electronic supplementary material.

Supplementary Material 1

Supplementary Material 2

Supplementary Material 3

## Data Availability

The data presented in this study are available on request from the corresponding author (N. Virji-Babul) due to restrictions related to the age of the participants.
