# Supplementary material for: Altered resting state EEG microstate dynamics in acute concussion in adolescents
Source: Sci Rep. 2026 Feb 2;16:6986. doi: 10.1038/s41598-026-37259-7 (PMC12921308; doi:10.1038/s41598-026-37259-7)
Supplement: Supplementary file 3 — Supplementary Material 3 [file 41598_2026_37259_MOESM3_ESM.docx]

The following analyses were conducted to determine the optimal number of clusters.


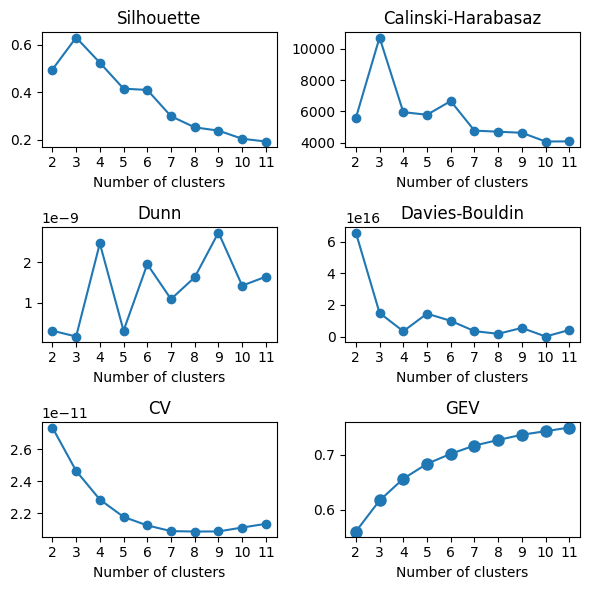


Figure S1: Results of six clustering evaluation metrics applied across a range of cluster numbers (2–11) to determine the optimal number of clusters. The top two metrics suggest three clusters as optimal, while the middle two indicate four as the optimal number. The cross-validation criterion identifies seven clusters as the elbow point. The GEV (Global Explained Variance) metric does not show a clear elbow.

Microstate Maps for 7 and 4 cluster solutions:

7- clusters:


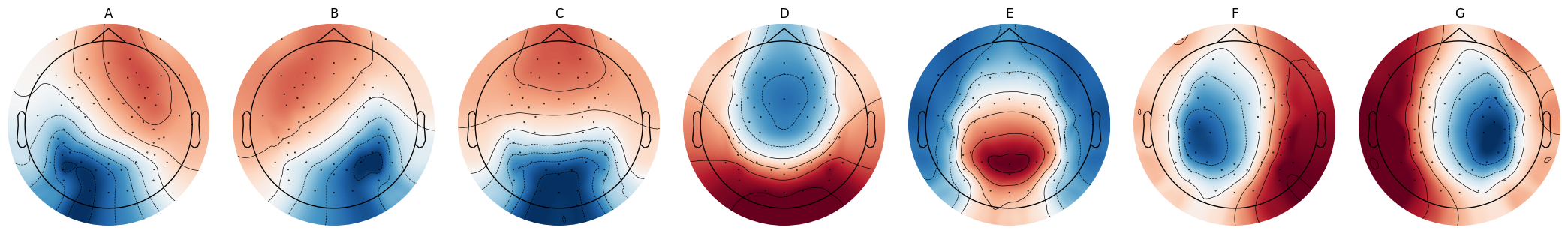


4-clusters:


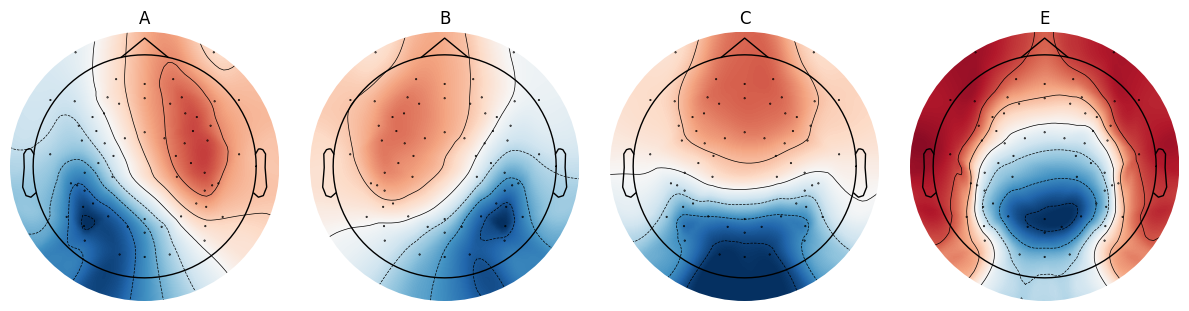


Groups separated:

7-clusters Control:


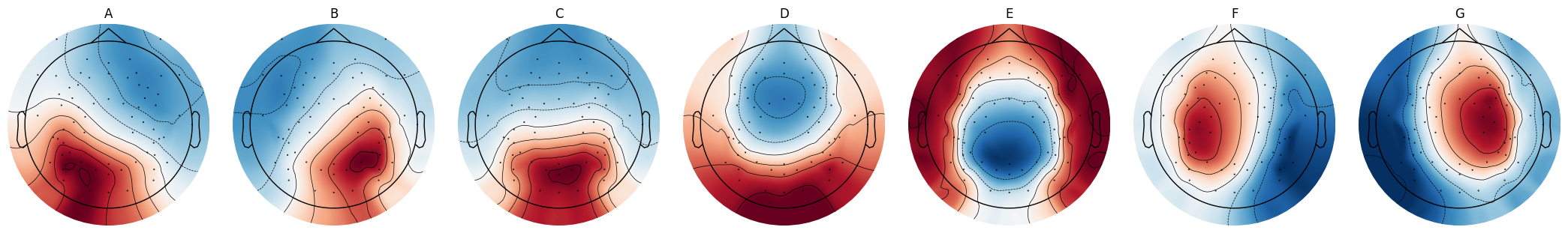


7_clusters mTBI:


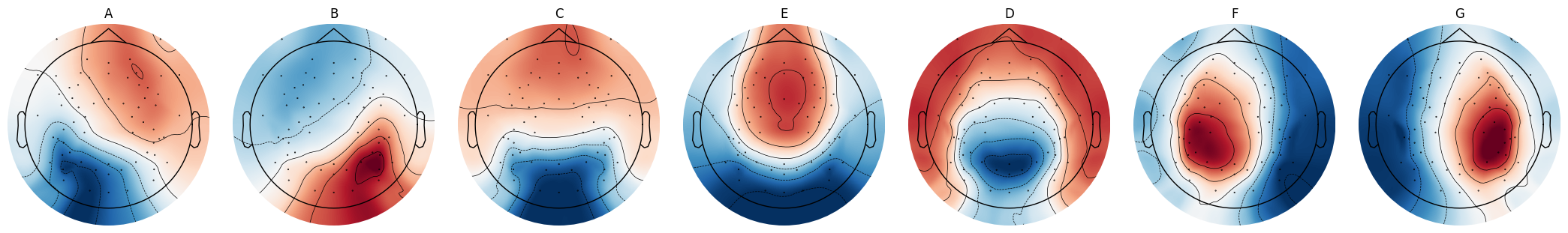


4-clusters Control:


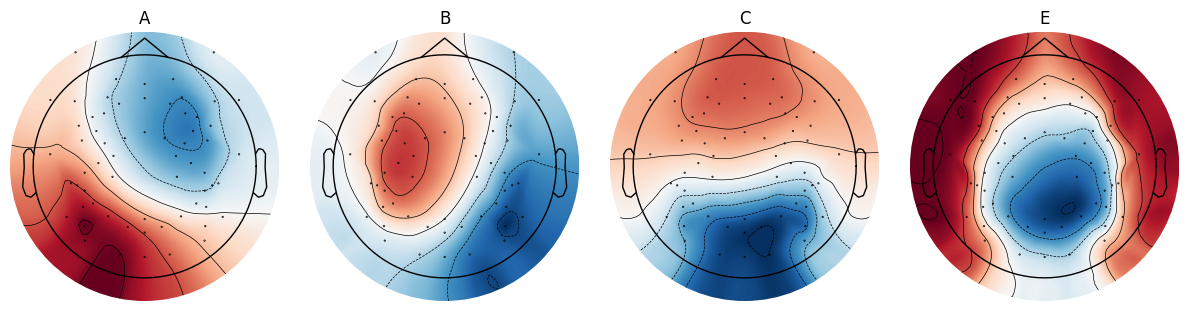


4-clusters mTBI:


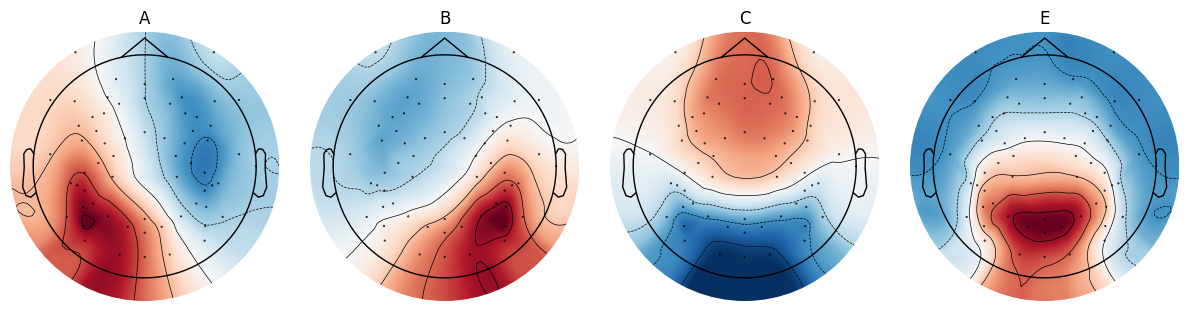


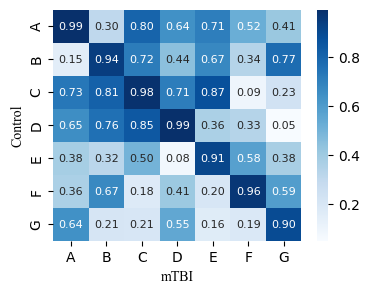


Figure S2: Heatmap showing correlation between microstate maps generated separately for the concussed and control groups. High correlations (>0.9) are observed between microstates with the same labels across groups which confirms that the microstates do not differ in topography between concussed and control participants. This supports the decision to generate the final set of microstate maps using the combined data from both groups.

Lastly, using a 4-cluster microstate solution, we confirmed that the group differences remained consistent. Microstate C showed higher duration, occurrence, and time coverage in the concussed group, while microstate E showed lower duration and time coverage compared to controls.
